# Impact of water consumption on renal function in the general population: a cross-sectional analysis of KNHANES data (2008–2017)

**DOI:** 10.1007/s10157-020-01997-3

**Published:** 2021-01-04

**Authors:** Jong Ah Lo, Jin Sun Kim, Min Jee Jo, Eun Jung Cho, Shin Young Ahn, Gang Jee Ko, Young Joo Kwon, Ji Eun Kim

**Affiliations:** 1 Department of Internal Medicine, Korea University Guro Hospital, Gurodong-ro 148, Guro-gu, Seoul, 08308 South Korea; 2 Department of Internal Medicine, Korea University College of Medicine, 150-7 Anamdong 5(o)-ga, Seongbuk-gu Seoul, 02841 South Korea

**Keywords:** Body fluid distribution, Body weight, Chronic kidney disease, Dehydration, Water intake

## Abstract

**Background:**

The renoprotective effect of water intake remains unclear. We aimed to investigate the relationship between water intake and renal impairment in the Korean general population, focusing on individual differences in body fluid distribution and risk of chronic dehydration.

**Methods:**

We conducted a cross-sectional analysis of the 2008–2017 Korea National Health and Nutrition Examination Survey (KNHANES). Adult participants who had body weight and serum creatinine data and had answered 24-h recall nutritional survey were included. Four water intake groups were defined by daily total water intake per body weight: lowest (< 20 mL/kg/day), low-moderate (20–29.9 mL/kg/day), high-moderate (30–49.9 mL/kg/day), and highest (≥ 50 mL/kg/day). We assessed the risk of renal impairment (estimated glomerular filtration rate ≤ 60 mL/min/1.73 m^2^) according to water intake.

**Results:**

In total of 50,113 participants, 3.9% had renal impairment. The risk of renal impairment gradually decreased as water intake increased. After adjustment of sodium intake, the trend of renoprotective effect was remained in low-moderate and high-moderate water intake group compared to low intake group, whereas no significant impact was observed with the highest water intake due to concurrent intake of high sodium. In subgroup analysis, the renoprotective effect of water intake was significant in the participants with elderly, male and daily sodium intake over 2 g/day.

**Conclusions:**

High daily water intake is renoprotective. Our data may provide an important basis for determining the amount of water intake needed to prevent renal impairment, considering variations in body weight, body composition and risk of chronic dehydration.

**Electronic supplementary material:**

The online version of this article (10.1007/s10157-020-01997-3) contains supplementary material, which is available to authorized users.

## Introduction

The number of people affected by chronic kidney disease (CKD) is substantially increasing [1]. In addition, patients with CKD have complications affecting various organs, and these complications increase the risk of death [2]. Accordingly, CKD constitutes one of the major financial burdens on public health systems; therefore, reducing the incidence of CKD and delaying its progression is a globally important medical challenge [1]. Multiple studies have identified risk and prognostic factors for CKD, and both lifestyle factors and medical comorbidities have been found to be important in CKD. Many global guidelines recommend lifestyle modifications such as salt restriction, low-protein diet, and regular exercise [3, 4].

Dehydration is classically considered a temporary and reversible state that is associated with acute renal dysfunction and does not cause long-term kidney complications [5]. However, chronic dehydration may be related to CKD, especially in agricultural communities where heat exposure is common due to climatic or geographical conditions [6]. It has been suggested that chronic dehydration may cause CKD by three potential mechanisms [5]. First, sustained high levels of vasopressin due to dehydration-related hyperosmolality induce morphological and functional changes in the kidney [7]. Second, dehydration-associated hyperosmolarity elevates metabolism of endogenous fructose, and the process requires considerable intracellular ATP consumption, leading to activation of AMP deaminase, oxidative stress, and the production of chemokines [8]. Third, dehydration induces hyperuricemia, which may contribute to CKD through vasculopathy, glomerular hypertension, and tubular injury [5, 9].

Several observational and prospective studies have evaluated the renoprotective effect of water intake in various conditions, such as the general population and patients with CKD [10–13]. However, it is still unclear whether water intake can contribute to the maintenance of kidney function, and there is no definite recommendation on the proper amount of water intake. Here, we aimed to investigate the association between water intake and renal impairment in the Korean population using the databases of the Korea National Health and Nutrition Examination Survey (KNHANES).

## Materials and methods

### Subjects

In this study, we used the databases of the fourth to seventh KNHANES, performed in 2007–2018. We excluded data from the first year (2007) of the fourth KNHANES and the last year (2018) of the seventh KNHANES because of limited data. Therefore, we ultimately assessed data from a 10-year period (2008–2017). The KNHANES is a national, population-based, cross-sectional survey collected annually by a governmental organization, the Korea Centers for Disease Control and Prevention (KCDC), to monitor public health. The KNHANES employs a stratified, multistage, probability sampling design based on geographic area, gender, and age group.

**Fig. 1:**
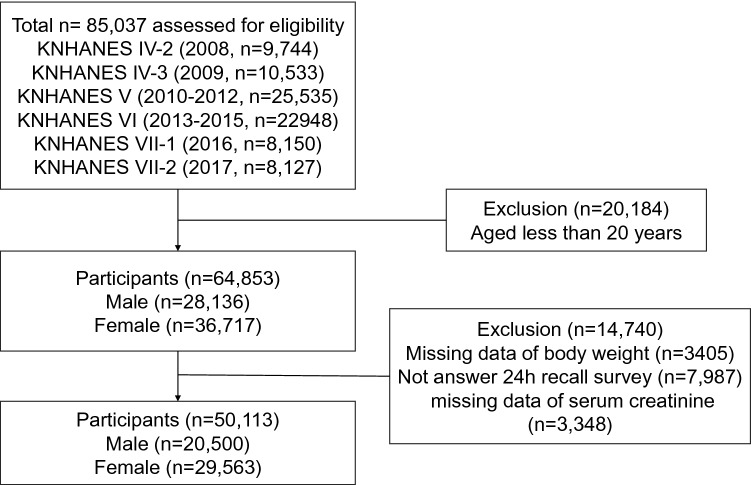
Flow chart of study participant selection. KNHANES, Korean National Health and Nutritional Examination Survey

Of the 85,037 participants who were enrolled during the 10-year span of this dataset, we finally included 50,113 in this study, excluding 20,184 participants under 20 years old as well as 14,739 participants who had missing data on body weight, laboratory results including creatinine, or no responses to a 24-h recall nutritional survey including information about water intake. The detailed inclusion and exclusion processes are shown in Fig. 1.

### Study design and data collection

This study was conducted as a cross-sectional analysis with a large-scale population. We collected demographic and socioeconomic characteristics, including age, gender, education status, household income, occupational type, working hours, frequency of alcohol consumption, smoking status, height, body weight, and body mass index. The participants were divided into three occupational types according to employment status and occupational category: inoccupation, manual labor and nonmanual labor. Additionally, the participants’ medical histories were collected, including hypertension and diabetes.

The amount of water intake and sodium intake in a day was assessed by the 24-h recall method, which is an open-label nutritional survey method for estimating food intake. In this study, two items related to water intake were combined as total daily water intake. First, the amount of mineral water or diluted tea as a substitute for mineral water was specified as the number of cups (where 1 cup = 200 mL). Second, the total amount of fluid in all foods, including beverages, was calculated using the food composition table created and validated by the Rural Development Administration. The detailed protocol for the nutrition survey is presented on the KNHANES website [14].

Finally, the participants were classified into 4 groups according to their total daily water intake per body weight: lowest water intake, < 20 mL/kg/day; low-moderate water intake, 20–29.9 mL/kg/day; high–moderate water intake, 30–49.9 mL/kg/day; highest water intake, ≥ 50 mL/kg/day.

### Study outcome

Although the diagnosis of renal impairment in clinical practice is based on the finding of a decrease in the glomerular filtration rate (GFR) and/or on the detection of elevated urinary excretion of albumin [3], we defined renal impairment as the estimated GFR (eGFR) ≤ 60 mL/min/1.73 m^2^, as in other studies, due to lack of urinary microalbumin concentration data during 2008–2010 and 2015–2017 [15, 16].

Serum creatinine was measured by a colorimetric method (ADVIA1650, Siemens, USA) before 15 February 2008 and by the Jaffe rate-blanked and compensated method (Hitachi Automatic Analyzer 7600, Hitachi, Tokyo, Japan) after 20 February 2008. eGFR was calculated using the Chronic Kidney Disease Epidemiology Collaboration (CKD-EPI) equation [17].

### Statistical analyses

Continuous variables were presented as the mean ± standard deviation (SD) or median (interquartile range) depending on the normality of the distribution, and categorical variables were presented as the number and percentage. Among the water intake groups, differences in continuous variables were analyzed by one-way analysis of variance or the Kruskal–Wallis test, and differences in categorical variables were analyzed by the Chi-squared test. Odds ratios (ORs) and 95% confidence intervals (CIs) for renal impairment were calculated using logistic regression analysis. In multivariable analysis, the baseline characteristics deemed to be possible confounding factors were adjusted in order: Model 1 included age and gender; Model 2 included age, gender, body mass index and comorbidities (hypertension and diabetes); Model 3 included all variables in model 2 and socioeconomic characteristics (alcohol consumption, smoking status, education status, household income, occupational type and working hours); Model 4 included all variables in model 3 and total daily sodium intake. Age was presented and adjusted as a categorical variable by 10 years.

Additionally, we conducted subgroup analysis according to age, gender and total daily sodium intake for renal impairment with adjustment for other risk factors and plotted the results as a forest plot. All statistical analyses were performed by Stata/SE ver. 15.1 (Stata Co., College Station, Texas, USA).

## Results

### Baseline characteristics

The total number of participants was 50,113, with 29,563 females (59.0%). There were 23,573 (47.0%) participants under the age of 50 years, while octogenarians were the least frequent age category, accounting for 1391 (2.8%) participants. The median daily water intake of all patients was 1.8 L/day, and the mean eGFR was 93.4 ml/min/1.73 m^2^. The overall prevalence of renal impairment was 3.9%. As the amount of water intake increased, the prevalence of renal impairment gradually decreased: lowest, 6.9%; low-moderate, 4.1%; high-moderate, 2.8%; and highest, 1.8%.

Those with the highest water intake were younger (under 50 years, 38.1 vs. 55.1%), less likely to be female (64.4 vs. 54.8%), more likely to complete education beyond high school (46.9 vs. 73.3%), more likely to have high household income (high-moderate income and above, 41.5 vs. 63.8%), more likely to have nonmanual labor occupations (23.8 vs. 38.8%), more frequent consumers of alcohol (2 or more times per month, 30.8 vs. 52.3%), more likely to be current smokers (15.4 vs. 24.8%) and less likely to have hypertension (38.1 vs. 22.7%) or diabetes (13.6 vs. 8.1%) than those with the lowest intake. Higher water intake was associated with higher sodium intake. The baseline characteristics of the participants are detailed in Table 1.

**Table 1:** Baseline characteristics of the overall and water intake stratified participants

	All participant (*n* = 50,113)	Total daily water intake per body weight			
		Lowest < 20 mL/kg/day (*n* = 9907)	Low-moderate 20–29.9 mL/kg/day (*n* = 15,946)	High-moderate 30–49.9 mL/kg/day (*n* = 19,161)	Highest ≥ 50 mL/kg/day (*n* = 5099)
Female sex, %	29,563 (59.0)	6375 (64.4)	9468 (59.4)	10,928 (57.0)	2792 (54.8)
Age, years					
20–29	5287 (10.6)	834 (8.4)	1641 (10.3)	2107 (11.0)	705 (13.8)
30–39	8919 (17.8)	1487 (15.0)	2739 (17.2)	3690 (19.3)	1003 (19.7)
40–49	9367 (18.7)	1460 (14.7)	2881 (18.1)	3923 (20.5)	1103 (21.6)
50–59	9588 (19.1)	1630 (16.5)	2922 (18.3)	3900 (20.4)	1136 (22.3)
60–69	8970 (17.9)	1854 (18.7)	2961 (18.6)	3365 (17.6)	790 (15.5)
70–79	6591 (13.2)	2085 (21.1)	2305 (14.5)	1877 (9.8)	324 (6.4)
≥ 80	1391 (2.8)	557 (5.6)	497 (3.1)	299 (1.6)	38 (0.8)
Household income, %					
Low	9891 (19.7)	3068 (31.0)	3352 (21.0)	2858 (14.9)	613 (12.0)
Low-moderate	12,546 (25.0)	2617 (26.4)	4078 (25.6)	4662 (24.3)	1189 (23.3)
High-moderate	13,470 (26.9)	2268 (22.9)	4290 (26.9)	5432 (28.4)	1480 (29.0)
High	13,716 (27.4)	1843 (18.6)	4059 (25.5)	6038 (31.5)	1776 (34.8)
Education level, %					
≤ Elementary school	12,343 (24.6)	3781 (38.2)	4253 (26.7)	3637 (19.0)	672 (13.2)
Middle school	5361 (10.7)	1059 (10.7)	1743 (10.9)	2048 (10.7)	511 (10.0)
High school	15,714 (31.4)	2623 (26.5)	4892 (30.7)	6339 (33.1)	1860 (36.5)
≥ College or graduate school	14,891 (29.7)	2024 (20.4)	4469 (28.0)	6523 (34.0)	1875 (36.8)
Occupational type, %					
Inoccupation	19,878 (39.7)	4601 (46.4)	6457 (40.5)	7158 (37.4)	1662 (32.6)
Manual labor	12,536 (25.0)	2517 (25.4)	4036 (25.3)	4715 (24.6)	1268 (24.9)
Non-manual labor	15,804 (31.5)	2358 (23.8)	4836 (30.3)	6632 (34.6)	1978 (38.8)
Working hours (h)	42.2 ± 18.6	41.9 ± 19.8	42.1 ± 19.0	42.3 ± 18.0	42.4 ± 18.1
Frequency of alcohol consumption (per month), %					
Never	6649 (13.3)	1971 (19.9)	2176 (13.7)	2074 (10.8)	428 (8.4)
≤ 1	22,025 (44.0)	4605 (46.5)	7247 (45.5)	8264 (43.1)	1909 (37.4)
2–4	10,357 (20.7)	1666 (16.8)	3283 (20.6)	4257 (22.2)	1151 (22.6)
> 4	9999 (20.0)	1382 (14.0)	2878 (18.1)	4224 (22.0)	1515 (29.7)
Smoking status, %					
Non-smoker	30,097 (60.1)	6289 (63.5)	9769 (61.3)	11,268 (58.8)	2771 (54.3)
Ex-smoker	10,201 (20.4)	1907 (19.3)	3242 (20.3)	4049 (21.1)	1003 (19.7)
Current smoker	9073 (18.1)	1527 (15.4)	2690 (16.9)	3594 (18.8)	1262 (24.8)
Height (cm)	161.9 ± 9.2	160.2 ± 9.4	161.7 ± 9.3	162.6 ± 9.0	162.9 ± 8.8
Body weight (kg)	62.5 ± 11.7	63.8 ± 12.5	63.2 ± 11.8	62.0 ± 11.2	59.9 ± 10.7
BMI categories, %					
Underweight (< 18.5 kg/m^2^)	2043 (4.1)	258 (2.6)	499 (3.1)	889 (4.6)	397 (7.8)
Normal (18.5–22.9 kg/m^2^)	19,733 (39.4)	3019 (30.5)	5817 (36.5)	8254 (43.1)	2643 (51.8)
Overweight (23–24.9 kg/m^2^)	11,844 (23.6)	2278 (23.0)	3819 (24.0)	4639 (24.2)	1108 (21.7)
Obese (25–29.9 kg/m^2^)	14,329 (28.6)	3543 (35.8)	5032 (31.6)	4879 (25.5)	875 (17.2)
Severe obese (≥ 30 kg/m^2^)	2146 (4.3)	802 (8.1)	773 (4.9)	497 (2.6)	74 (1.5)
Hypertension, %	15,566 (31.1)	3779 (38.1)	5234 (32.8)	5396 (28.2)	1157 (22.7)
Diabetes mellitus, %	5413 (10.8)	1347 (13.6)	1839 (11.5)	1815 (9.5)	412 (8.1)
Renal impairment (eGFR ≤ 60 ml/min/1.73 m^2^), %	1961 (3.9)	685 (6.9)	660 (4.1)	526 (2.8)	90 (1.8)
eGFR (ml/min/1.73 m^2^)	93.4 ± 17.6	89.7 ± 19.3	92.6 ± 17.7	95.0 ± 16.7	97.6 ± 15.6
eGFR ≥ 90, %	30,198 (60.3)	5057 (51.0)	9260 (58.1)	12,262 (64.0)	3619 (71.0)
eGFR 60–89, %	17,954 (35.8)	4165 (42.0)	6026 (37.8)	6373 (33.3)	1390 (27.3)
eGFR 30–59, %	1817 (3.6)	634 (6.4)	612 (3.8)	488 (2.6)	83 (1.6)
eGFR 15–29, %	103 (0.2)	34 (0.3)	33 (0.2)	30 (0.2)	6 (0.1)
eGFR < 15, %	41 (0.1)	17 (0.2)	15 (0.1)	8 (0.0)	1 (0.0)
Total daily water intake (ml/day)	1812.8 (1325.4–2457.6)	971.1 (792.6–1160.5)	1543.3 (1341.0–1778.5)	2291.9 (1960.1–2675.8)	3446.9 (2994.2–4088.7)
Total daily sodium intake (g/day)	3.6 (2.4–5.4)	2.6 (1.7–3.9)	3.4 (2.2–5.0)	4.1 (2.8–6.1)	4.9 (3.6–7.2)

*BMI* body mass index, *eGFR* estimated glomerular filtration rate

### Prevalence of renal impairment according to water intake amount

**Fig. 2:**
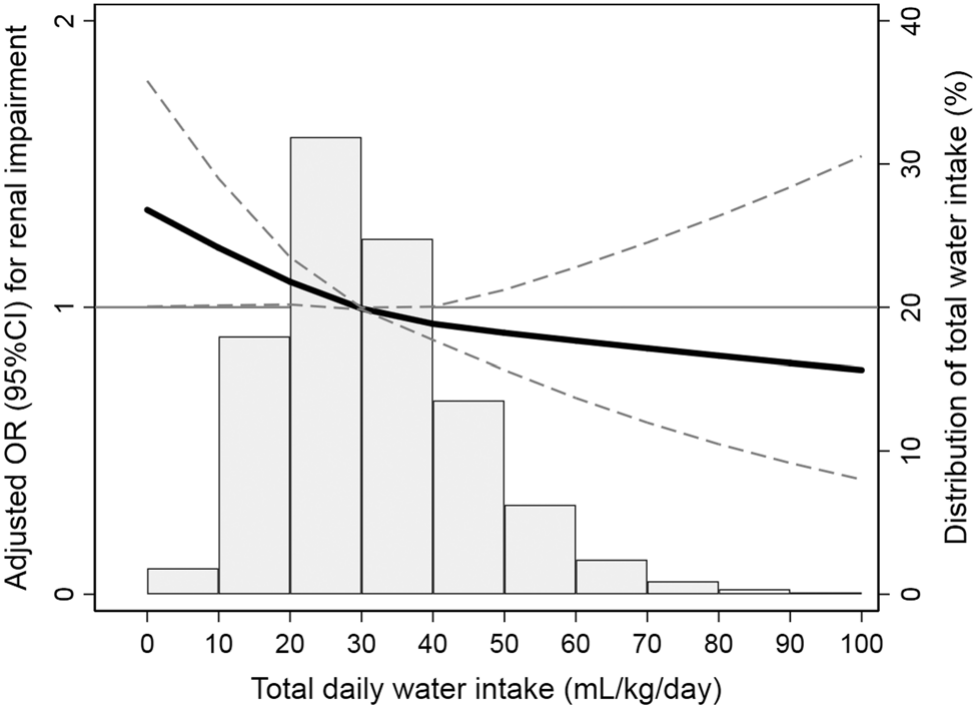
Restricted cubic spline curve showing adjusted odds ratios (solid line) and 95% confidence intervals (dashed lines) for renal impairment associated with total daily water intake. The reference value for the odds ratio is the median of the amount of water intake per kilogram of body weight. Covariates included age, gender, BMI, hypertension, diabetes mellitus, education status, household income, occupational type and total daily sodium intake

Compared with the lowest water intake group, the ORs for renal impairment gradually decreased as daily total water intake increased (low-moderate water intake, OR 0.58, 95% CI 0.52–0.65; high-moderate water intake, OR 0.38, 95% CI 0.34–0.43; highest water intake, OR 0.24, 95% CI 0.19–0.30) (Table 2). All demographic, socioeconomic, and medical history characteristics and sodium intake significantly affected the prevalence of renal impairment. The inverse relationship between total water intake and renal impairment remained significant after adjusting for the abovementioned risk factors according to models 1, 2 and 3. However, only the participants with low-moderate and high-moderate water intake had a significantly lower risk of renal impairment than those with the lowest water intake after additional adjustment for sodium intake according to model 4. Figure 2 shows a restricted cubic spline curve of the ORs of total daily water intake for renal impairment.

**Table 2:** Association between total daily water intake per body weight and renal impairment (eGFR ≤ 60 ml/min/1.73 m^2^)

Total daily water intake per body weight	Renal impairment, %	OR (95% CI)				
		Univariable	Model 1	Model 2	Model 3	Model 4
Lowest (< 20 mL/kg/day)	685 (6.9)	Reference	Reference	Reference	Reference	Reference
Low-moderate (20–29.9 mL/kg/day)	660 (4.1)	0.58 (0.52–0.65)	0.78 (0.69–0.88)	0.78 (0.69–0.89)	0.82 (0.71–0.95)	0.86 (0.75–0.99)
High-moderate (30–49.9 mL/kg/day)	526 (2.8)	0.38 (0.34–0.43)	0.67 (0.60–0.76)	0.73 (0.64–0.83)	0.75 (0.64–0.87)	0.81 (0.69–0.95)
Highest (≥ 50 mL/kg/day)	90 (1.8)	0.24 (0.19–0.30)	0.56 (0.44–0.71)	0.66 (0.52–0.84)	0.69 (0.52–0.92)	0.76 (0.57–1.02)

Model 1: adjustment for age and gender

Model 2: adjustment for age, gender, BMI, HTN, and diabetes

Model 3: adjustment for age, gender, BMI, HTN, diabetes, household income, education level, occupational type, working hours, frequency of alcohol consumption, and smoking status

Model 4: adjustment for age, gender, BMI, HTN, diabetes, household income, education level, occupational type, working hours, frequency of alcohol consumption, smoking status and total daily sodium intake

*eGFR* estimated glomerular filtration rate, *OR* odds ratio, *CI* confidence interval, *BMI* body mass index, *HTN* hypertension

### Association of water intake and renal impairment according to subgroups

**Fig. 3:**
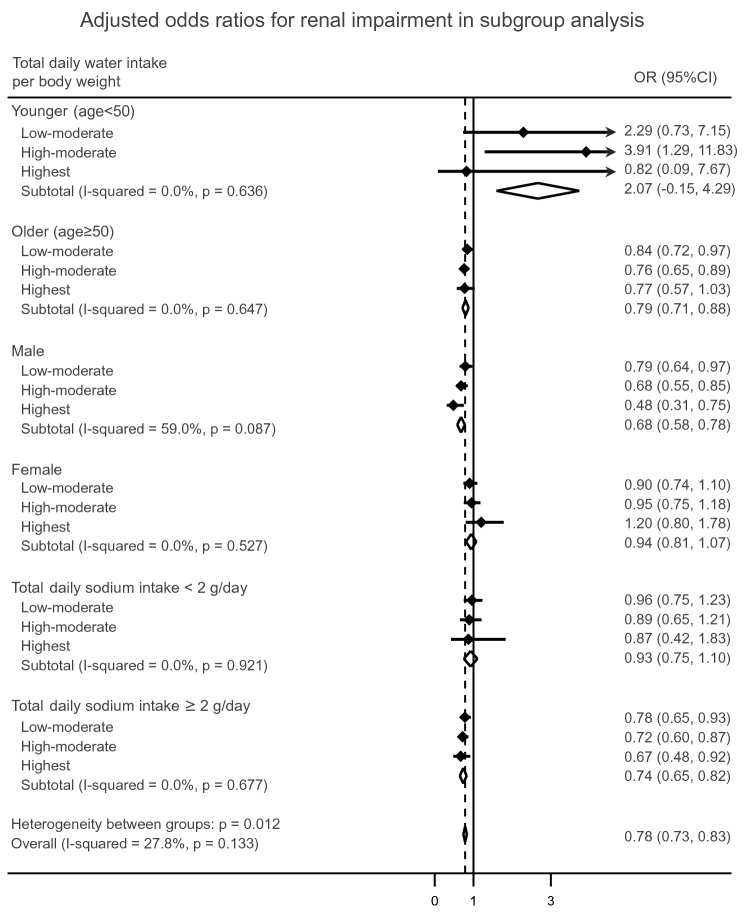
Subgroup analysis results. The adjusted odds ratios and 95% confidence intervals were calculated in the multivariable logistic analysis adjusted for age, gender, BMI, hypertension, diabetes mellitus, education status, household income, occupational type and total daily sodium intake. Black diamonds indicate odds ratios, and error bars indicate 95% confidence intervals. A solid vertical line represents no effect, and a dashed vertical line represents an overall effect of water intake

In the subgroup analysis, the association between water intake and renal impairment was different for age, gender and daily sodium intake (Fig. 3). In the group aged under 50 years, there was no consistent relationship between water intake and renal impairment. However, in the group aged 50 years or over, the prevalence of renal impairment was significantly lower with low-moderate and high-moderate water intake compared with the lowest water intake. In a subgroup analysis based on gender, the protective effect of water intake on renal function was detected only in male participants (low-moderate water intake, adjusted OR 0.79, 95% CI 0.64–0.97; high-moderate water intake, adjusted OR 0.68, 95% CI 0.55–0.85; highest water intake, adjusted OR 0.48, 95% CI 0.31–0.75). In the group with sodium intake ≥ 2.0 g/day, the adjusted ORs (95% CIs) were 0.78 (0.65–0.93), 0.72 (0.60–0.87) and 0.67 (0.48–0.92) with low-moderate, high-moderate, and highest water intake, respectively. However, there was no association between water intake and renal impairment in the group with sodium intake < 2.0 g/day.

## Discussion

In our study, the risk of renal impairment gradually decreased as daily total water intake per body weight increased, even though the effect was attenuated after adjustment for confounding factors. The kidney is the main organ that regulates body fluid balance and filters out uremic toxins [18]. An increase in water intake increases the clearance of sodium, urea and osmolytes [19], while deficient water intake increases the concentration of urine, and higher urine concentration may contribute to glomerular hyperfiltration and the development of albuminuria [20]. Additionally, when exposed to chronic deficiency of vascular fluid volume, the kidneys are highly susceptible to subclinical damage, and cumulative damage from repeated insults can accelerate the development of CKD [5, 7].

Several observational studies regarding the association between renal dysfunction and fluid intake have been reported. Two Australian cross-sectional studies showed an inverse relationship between total fluid intake and the prevalence of CKD [10]. In another cross-sectional study in US, the highest quintile of fluid intake was associated with an imprecise and nonsignificant risk of CKD compared with the lowest quintile of fluid intake [11]. Additionally, this US study examined renal outcomes relating to drinking plain water versus other fluids, and benefits were seen only for the intake of plain water, with no effects for the increased intake of other beverages. Meanwhile, a prospective cohort study in Australia with an old age population showed no significant association of daily fluid intake and the occurrence of CKD [12]. However, the study only assessed fluids in foods and other beverages, not in plain water.

Compared to these previous studies, the present study showed a positive relationship between water intake and renal function. We assessed the full amount of water intake for analysis and presented the body weight-adjusted water amount rather than the water volume itself as the important variable associated with kidney. Body fluid volume was traditionally calculated and estimated using body weight, in light of the strong correlation between these variables [21]. Therefore, we assumed that the required amount of fluid could also vary depending on the body weight, and regarding the results of our study, we propose that daily water intake of more than 10 ~ 20 ml per body weight may have a protective effect on the kidney. We additionally performed the same analysis using the water intake variable corrected for the body surface area and showed similar results with body weight-adjusted water intake in the univariate analysis, but after adjustment for confounders, the importance decreased (Supplementary Table 1).

The median unadjusted volume of water intake was 1.81 L/day, which is considerably lower than the reported water intake volumes in previous observational studies, including 2.45 L/day in an Australian study and 2.9 L/day in a US study. In the US, Institute of Medicine recommendations from 2004 set adequate levels for total water intake from all foods and liquids at 3.7 L for men and 2.7 L for women [22]. However, in our data, only 8.2% of men and 11.5% of women met these recommendations. This difference may be due to various diet and lifestyle differences between countries or ethnic groups, but the difference in body shape, weight, and composition is considered a key cause. Considering this, it seems more reasonable to make recommendations with weight-adjusted values than simply to present a water intake in terms of volume without considering body composition.

The higher water intake group tended to be younger, which is consistent with other observational studies [11, 12]. Water intake was associated with renal impairment in participants ≥ 50 years but not in younger participants. These results may be related to changes in the body fluid distribution and the response to water deprivation with age [23, 24]. Equal amounts of fluid loss represent more severe dehydration in the elderly than in younger people due to an impaired ability to conserve water and a decrease in the percent total body water with advanced age [23]. These age-dependent differences are presumed to be the cause of the higher association between water intake and renal function in older people than in younger people.

Similar to other studies, we reported an increasing proportion of males according to water intake increase [11, 12]. Additionally, we found a distinctive association between water intake and renal function in males but not in females (Fig. 3). In our data, the proportion of manual labor workers was higher and working hours were longer in male participants than in female participants (manual labor worker 35.7% vs. 17.6%; working hours 38.7 ± 18.7 vs. 45.8 ± 17.9 h), suggesting the possibility of a high risk of chronic dehydration in males rather than females. In addition, we suggest the possibility that the difference in fat and muscle distribution between women and men influenced the response to water intake.

Interestingly, the participants in the highest water intake group no longer had a significantly lower risk of renal impairment after adjusting for sodium intake. Sodium, a main extracellular ion, acts as the main determinant of osmolality and water movement [25]. The relationship between excessive sodium loading and deterioration of kidney function is well known [25, 26]. As shown in Table 1, water intake showed a quantitatively positive relationship with sodium intake. The median total daily sodium intake of those with the highest water intake was 4.9 g/day, nearly 2.5 times the recommended amount and higher than the reported sodium intake of the highest water intake groups in previous observational studies, including 2.6 g/day in an Australian study and 4.7 g/day in a US study [11, 12]. Therefore, regarding the reason for the loss of significance after sodium adjustment in the highest water intake group, we suggest the hypothesis that the detrimental effect of high sodium intake might exceed the renoprotective property of water intake in the highest water intake group. In addition, we also found a differential association between water intake and renal function in subgroup analysis by amount of sodium intake. According to these findings, we suggest that water and sodium are closely related in fluid homeostasis and may affect renal function in a manner dependent on each other.

This study has several limitations. First, based on cross-sectional surveys, we could not distinguish whether the renal impairment was acute or chronic. There were no data on whether the participants received maintenance dialysis or restricted their water intake due to edema. However, only 144 (0.3%) participants had an eGFR < 30 ml/min/1.73 m^2^, which was negligible. Second, a 24-h nutritional dietary recall method may not accurately reflect the usual dietary behavior and food preference of individuals. Nonetheless, the 24-h dietary recall survey has been validated and extensively utilized as the most popular method of large-scale nutritional surveys, and a high completion rate could be achieved due to convenience (77,4250 of 85,037 participants (88.8%)). Third, we could not distinguish the origin of water intake in detail. In other studies, the association between water intake and renal outcome appears to be modified by the source of water [27–29]. There is some evidence that fructose can cause kidney damage, and excessive sweetener intake may increase the risk of obesity or diabetes, an established risk factor for CKD [27]. Several large observational studies have demonstrated that high intake of sugar or carbonated beverages is linked to albuminuria and CKD [28, 29]. Further studies about the difference in the effect of water intake according to the source of food are warranted. Last, although the KNHANES survey was performed throughout all seasons each year, we could not assess the seasonal effect on outcomes due to a lack of data. Korea has four distinct seasons with dynamic temperatures and humidity, and the manual labor workers in our study accounted for 25% of the total participants. Thus, further studies should be considered to investigate whether this relationship between water intake and kidney is affected by seasons or climates.

Water is an essential nutrient for life [30]. The importance of water intake in public health is often underestimated with relatively small numbers of research compare to other nutrients or medicines. According to the present study, daily fluid intake is closely related to kidney and dehydration may increase the risk of renal dysfunction. We also suggest that higher daily water intake per body weight may have a renoprotective effect, especially in older and male populations. The results of this Korean general population-based survey may be an important basis for determining water intake recommendations in specific populations to prevent renal impairment.

## Electronic supplementary material

Below is the link to the electronic supplementary material.

Supplementary file 1 (DOCX 18 KB)
