# Supplementary material for: Impact of water consumption on renal function in the general population: a cross-sectional analysis of KNHANES data (2008–2017)
Source: Clin Exp Nephrol. 2021 Jan 4;25(4):376–84. doi: 10.1007/s10157-020-01997-3 (PMC7966133; doi:10.1007/s10157-020-01997-3)
Supplement: Supplementary file 1 — (DOCX 18 KB) [file 10157_2020_1997_MOESM1_ESM.docx]

| **Supplementary table 1. Association between total daily water intake per BSA and renal impairment (eGFR ≤ 60ml/min/1.73m^2^)** | | | | | | |
| --- | --- | --- | --- | --- | --- | --- |
| Total daily water intake  per BSA | Renal impairment, % | OR (95% CI) | | | | |
|  |  | Univariable | Model 1 | Model 2 | Model 3 | Model 4 |
| Lowest  (<500 mL/m^2^/day) | 206 (8.0) | Reference | Reference | Reference | Reference | Reference |
| Low-moderate  (500-999.9 mL/m^2^/day) | 948 (5.2) | 0.63  (0.54-0.74) | 0.95  (0.80-1.12) | 0.98  (0.82-1.18) | 1.02  (0.83-1.26) | 1.06  (0.86-1.31) |
| High-moderate  (1000-1999.9 mL/m2/day) | 751 (2.9) | 0.34  (0.29-0.40) | 0.78  (0.66-0.93) | 0.81  (0.67-0.98) | 0.84  (0.68-1.05) | 0.92  (0.74-1.15) |
| Highest  (≥200 mL/m^2^/day) | 55 (1.6) | 0.19  (0.14-0.26) | 0.64  (0.47-0.88) | 0.68  (0.49-0.95) | 0.71  (0.48-1.06) | 0.81  (0.54-1.20) |
| Model 1: adjustment for age and gender Model 2: adjustment for age, gender, BMI, HTN, and diabetes Model 3: adjustment for age, gender, BMI, HTN, diabetes, household income, education level, occupational type, working hours, frequency of alcohol consumption, and smoking status  Model 4: adjustment for age, gender, BMI, HTN, diabetes, household income, education level, occupational type, working hours, frequency of alcohol consumption, smoking status and total daily sodium intake Abbreviations: BSA, body surface area; eGFR estimated glomerular filtration rate; OR, odds ratio; CI, confidence interval; BMI, body mass index; HTN, hypertension | | | | | | |
